# A comparison of individual and collective decision making for standard gamble and time trade-off

**DOI:** 10.1007/s10198-019-01155-x

**Published:** 2020-01-04

**Authors:** Arthur E. Attema, Han Bleichrodt, Olivier l’Haridon, Stefan A. Lipman

**Affiliations:** 1 Erasmus School of Health Policy and Management, Erasmus University, P. O. Box 1738, 3000 DR Rotterdam, The Netherlands; 2 Erasmus School of Economics, Erasmus University, Rotterdam, The Netherlands; 3 Research School of Economics, Australian National University, Canberra, Australia; 4 CREM, Université de Rennes 1, Rennes, France

**Keywords:** Collective decision making, Health state valuation, Standard gamble, Time trade-off, D90, I10, I19

## Abstract

Quality-Adjusted Life-Years (QALYs) are typically derived from individual preferences over health episodes. This paper reports the first experimental investigation into the effects of collective decision making on health valuations, using both time trade-off (TTO) and standard gamble (SG) tasks. We investigated collective decision making in dyads, by means of a mixed-subjects design where we control for learning effects. Our data suggest that collective decision making has little effect on decision quality, as no effects were observed on decision consistency and monotonicity for both methods. Furthermore, QALY weights remained similar between individual and collective decisions, and the typical difference in elicited weights between TTO and SG was not affected. These findings suggest that consulting with others has little effect on health state valuation, although learning may have. Additionally, our findings add to the literature of the effect of collective decision making, suggesting that no such effect occurs for TTO and SG.

**Electronic supplementary material:**

The online version of this article (10.1007/s10198-019-01155-x) contains supplementary material, which is available to authorized users.

## Introduction

Many decisions about health are made in deliberation with others, e.g. children, spouses or medical professionals. This collective feature of decisions about health is, however, not typically reflected in health outcomes research focused on Quality-Adjusted Life-Years (QALYs). The weights representing quality of life, that are required to calculate these QALYs (i.e. QALY weights), are typically determined through choice-based methodologies [1], such as standard gamble (SG) or time trade-off (TTO). Both methods are applied to the individual case, through decisions about one’s own (hypothetical) health outcomes [2, 3], i.e. no deliberation with others is allowed. As is well-documented in the health economic literature, QALY weights usually differ between SG and TTO [4–6]. SG weights are typically higher than TTO weights, and conventionally, this difference between SG and TTO was explained as resulting from deviations from the linear QALY model and expected utility (EU) theory which have both been found to be descriptively inaccurate [7–9]. Although it may be possible to measure these deviations and correct for their influences in SG and TTO [10], currently no consensus exists on how these biases^1^ are best measured or corrected for. Hence, the main motivation of this paper is to explore if the quality and outcomes of SG and TTO are affected by asking individuals to complete these tasks in groups, and if the difference between SG and TTO weights is reduced as a result.

The extant literature for monetary outcomes provides some indication that allowing individuals to discuss these complex decisions about health with others may be helpful. For example, collective decision making has been associated with less discounting and fewer time inconsistencies [11]. Other existing work on the effects of collective decision-making gives less firm results, with mixed evidence being reported for risk aversion [12–16], ambiguity aversion [13, 17, 18] and the violation rate of EU [19–21]. When effects of collective decision making occur, they are hypothesized to result from the deliberation, bargaining and exchange of information that takes place when deciding collectively (e.g. [14, 19]). Taken together, these studies suggest that risk preferences, which are relevant for SG, and time preferences, which are relevant for TTO, might be affected by collective decision making. For example, discounting of future life years leads to downwards bias in TTO [22–24], and if such discounting is lower in when individuals decide in a group [11] this could lead to higher TTO weights. Similarly, if groups are more willing to take risks [13], perhaps due to reduced overweighting of small probabilities of dying, this could yield lower SG weights. If such effects occur simultaneously, the difference between SG and TTO might reduce.

Only a few studies exist documenting effects of deliberation in groups or deciding collectively on SG and TTO weights. McIntosh et al. [25] found that completing SG in a panel and deliberating about responses decreased subsequent SG weights, and Karimi et al. [26] found that deliberation in a panel had an effect on individual TTO weights. Just a single study explored collective valuation for both SG and TTO and found only small effects [27]; however, this study used an anonymous voting system to obtain collective SG and TTO responses, i.e. deliberation between respondents was not allowed. Hence, those few studies on the effects of deliberation or collective decisions on QALY weights differ in several respects from the economic literature, in which typically smaller groups actually decide together (i.e. bargaining is included).

As such, we believe the evidence base on collective decision making precludes the formation of clear hypotheses for three reasons. First, next to the mixed evidence on risk preferences, an extensive psychological literature exists suggesting that in some cases detrimental effects of group decision making can be observed. This literature suggests that groups can engage in ‘groupthink’, which fosters limited information search and enhances confirmation bias [28, 29]. Second, the extant literature on collective decisions mostly studies monetary decision making, while SG and TTO involve health-related decision making, and these differ in many ways [30]. Third, those few available investigations on effects of collective decisions for health [25, 26, 31, 32] did not use an experimental design, i.e. often no control condition or comparator was in place. This complicates the interpretation of these studies’ findings, as these may be caused by learning instead (i.e. as a result of repeated measurement after deliberation). Indeed, it is well-known that such effects may occur in health state valuation (e.g. [33]). Hence, in our work we explore the effects of collective decisions for SG and TTO, whilst controlling for learning effects.

Our study adds to the earlier literature on collective decisions and health state valuation in several respects. We report the first experimental test of the effects of collective decision making on QALY weights, by using a control condition constructed to control for learning. More specifically, we obtained a baseline measurement for SG and TTO for each subject, after which we distinguished between groups and individuals for repeated decisions. By using such a control condition (similar to that of [17]), we are able to isolate the effect of deciding collectively on multiple facets of SG and TTO decisions (only related to deliberation, bargaining and information exchange). We explore if such effects of collective decisions exist on internal consistency criteria, and if SG and TTO weights change by deciding collectively. Importantly, we test if the difference between SG and TTO reduces, as this could indicate that the different biases that are suggested to produce this difference are reduced [22]. If that is the case, the use of collective decisions could provide an answer to the open questions surrounding the validity of QALY weights elicited with SG and TTO [34]. Finally, we test whether any possible effects of collective decision making carry over onto subsequent individual SG and TTO exercises for groups.

## Preliminaries

In this paper, we only consider chronic health profiles described as $\left( {Q, T} \right)$, with $Q$ denoting health status and $T$ denoting its duration in years. For brevity, we denote immediate death as $D$ and if health status is equal to full health (${\text{FH}}$) we write $Q = {\text{FH}}$. Under the assumption of completeness, decision makers are able to form preferences over health profiles, denoted using the conventional notation: $\succ$, ${ \succcurlyeq }$, and ${\sim }$ to represent strict preference, weak preference, and indifference, respectively. Most studies applying SG or TTO assume that decision makers form these preferences as modeled within the linear QALY model^2^ [35], i.e.:

1 $$V\left( {Q,T} \right) = U\left( Q \right) \times T.$$

Decision makers decide about health profiles, either under certainty (in case of TTO) or under risk (in case of SG). Risk is operationalized by presenting decision maker with lotteries of the following form: $\left( {Q, T} \right)_{p} \left( {Q^{\prime}, T^{\prime}} \right)$, which signifies that health profile $\left( {Q,T} \right)$ will be realized with probability $p$, and health profile $\left( {Q^{\prime}, T^{\prime}} \right)$ with probability $1 - p$.

The SG method involves determining probability *p* at which decision makers are indifferent between a sure outcome $\left( {Q, T} \right)$, and a risky prospect $\left( {{\text{FH}}, T} \right)_{p} \left( D \right)$. Probability $p$ is varied until the respondent is indifferent between a number of years ($T$) in health state $Q$ for certain and a gamble with two outcomes, which are ${\text{FH}}$ during the same time period ($T$), and $D$. These SG indifferences are typically evaluated under expected utility (EU) theory [36]. The TTO method, on the other hand, asks for a time equivalent in perfect health which yields indifference between $\left( {Q,T} \right)$ and $\left( {{\text{FH}}, T^{\prime}} \right)$, with $T > T^{\prime}$. The number of years $T^{\prime}$ is varied until the respondent is indifferent between $T$ years in health state $Q$ and $T^{\prime}$ years in ${\text{FH}}$. Given the assumptions listed above, and setting $U\left( {\text{FH}} \right) = 1$ and $U\left( D \right) = 0$ the SG indifference $\left( {{\text{Q}}, T} \right)\sim \left( {{\text{FH}}, T} \right)_{p} \left( D \right)$ is evaluated by $U\left( Q \right) \times T = p \times \left( {1 \times T} \right) + \left( {1 - p} \right) \times 0$, and, thus: $U\left( Q \right) = p$. The TTO indifference $\left( {Q, T} \right)\sim \left( {{\text{FH}}, T^{\prime}} \right)$ is evaluated by: $U\left( Q \right) \times T = 1 \times T^{\prime}$, and, thus, we obtain $U\left( Q \right)$ = $T^{\prime}/T$.

Bleichrodt [22] proposed that the typical differences between SG and TTO weights for the same health states may result from deviations from EU theory or the linear QALY framework, such as discounting, loss aversion and probability weighting. Thus, by evaluating SG and TTO without acknowledging these deviations, we should observe a gap between SG and TTO. Formally, we define the SG–TTO gap as the difference between $U\left( Q \right)$ as derived from SG and TTO: $\Delta \left( {{\text{SG}} - {\text{TTO}}} \right) = p - \left( {\frac{{T^{\prime}}}{T}} \right)$. We expect $\Delta \left( {{\text{SG}} - {\text{TTO}}} \right) > 0$, and explore if collective decision making has effects on the difference between SG and TTO. If that is the case this gap should decrease, which we test empirically in an experiment.

## Experiment

### Sample and design

A total of 163 Business Administration students (78 female, mean age 19.37, SD 1.57) participated in this experiment,^3^ which lasted around 55 min. Subjects were recruited via Erasmus Research Participation System, which rewards students with course credit for participation in scientific research. The experiment used a mixed between/within-subjects design (see Table 1) with two randomly assigned between-subjects conditions: individual decision making (IDM) and collective decision making (CDM). Experimental sessions were run on computers in sessions of two (CDM) or four (IDM) subjects sitting adjacently in separated cubicles. The experiment was programmed in Matlab, and instructions were provided on a separate sheet (see Online Appendix A). An instructor was present at all times to answer any questions subjects might have with regard to the procedure. Sessions consisted of three parts, with the experimental conditions IDM and CDM only differing in the second part. The first part served to establish a baseline measurement for SG and TTO weights, i.e. in Part 1 all subjects completed SG and TTO individually. In the second part, subjects in the CDM condition completed SG and TTO elicitations again collectively. Subjects in the IDM condition individually completed a filler task (adapted from [37]), which was not related to health states, risk or lotteries, to avoid confounding effects. The results of this filler task are not covered in this paper. In Part 3, to determine whether learning (IDM) or carryover effects (CDM) occurred, all subjects were presented with one final repetition of SG and TTO utility elicitation completed individually. When subjects finished Part 3, demographics were collected.

**Table 1:**
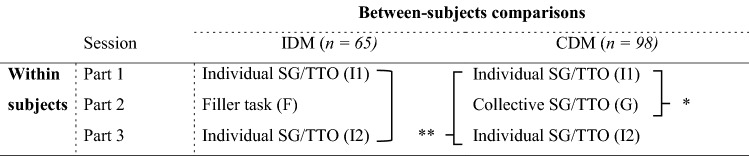
Overview experimental conditions

*The group effect, and **the carryover effect

### Measurements for SG and TTO

**Fig. 1:**
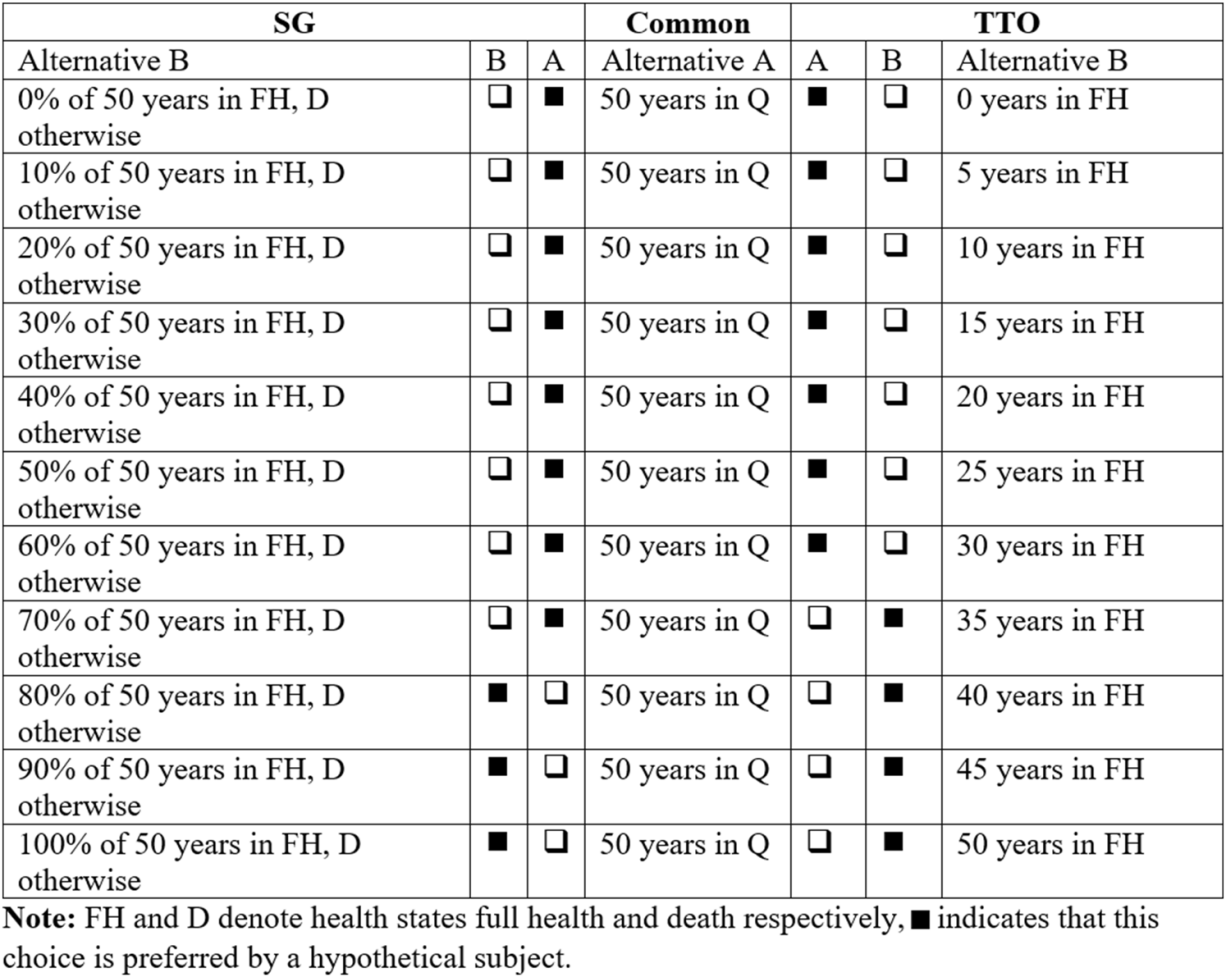
Example choice list for SG and TTO filled in by example participant. FH and D denote health states full health and death respectively, n indicates that this choice is preferred by a hypothetical subject

All SG and TTO elicitations were operationalized by using choice list methodology (see Online Appendix A for instructions and screenshots). This elicitation procedure, popularized by Holt and Laury [38], is used frequently for elicitation of risk and time preferences for monetary outcomes [39, 40]. Although we are not aware of any study using choice list methodology for SG and TTO, recently choice lists have also been used to elicit preferences for health outcomes (e.g. [41, 42]). Figure 1 shows a combined example of SG and TTO choice lists.

For choice lists based on the SG method, subjects were faced with a choice between two alternatives. Alternative A would make them certain to live 50 more years^4^ in some health state ($Q$), after which they would die. If they chose Alternative B, they would be taking a gamble. The following instruction was used to clarify the risk of Alternative B: ‘On the one hand, you have the chance $\left( {100 \times p\% } \right)$ of living 50 more years $\left( T \right)$ in full health (i.e. no problems on any dimension), after which you will die, but on the other hand, you have a chance $\left( {100 \times \left( {1 - p} \right)\% } \right)$ of dying within a week’. Subjects faced choice lists of 10 choices in which Alternative B varied; more specifically, $p$ increased. For each elicitation, a two-pronged approach was used. First, $p$ varied in increments of 10%, between 0 and 100%. After a switching point was obtained at this level, a second choice list was presented, which elicited a probability at the percentage point. For example, if a subject switched at $p$ = 80% in the first choice list (as in Fig. 1), she would face a second choice list that varied between 70 and 80% with increments of 1% (see Online Appendix B for screenshots).

For TTO choice lists, Alternative A was the same as for the SG choice lists, i.e. living 50 more years ($$T)$$ in the indicated health state ($$Q$$), followed by death. If subjects chose Alternative B, they would live $$T^{\prime}$$ more years in full health (i.e. no problems on any dimension), followed by death. A similar two-step elicitation procedure was in place, where, in the first choice list, $$T^{\prime}$$ varied between 0 and 50 years. In the second choice list, the indifference point of the first list was continued, and a more precise estimate was obtained by presenting subjects with a choice list with 10 increments of 0.5 year. For example, if a subject switched from A to B at $$T^{\prime} = 35$$ years (as in Fig. 1), she would face a choice list with Alternative B varying between 30 and 35 with 0.5 year increments (see Online Appendix B).

### Collective decision making task

If a session was randomized to be a CDM session it consisted of two subjects who arrived at the lab at the same time. Both subjects first completed Part 1 individually, i.e. the baseline measurement for SG and TTO, while seated in separate cubicles. After they were both finished with Part 1 (if necessary one of the subjects was asked to wait until the other was finished), subjects were asked to move to one of the adjacent cubicles together. In this cubicle, they were asked to repeat the task they just performed (Part 2) and instructed to freely discuss amongst each other until they reached an answer that was satisfactory for both of them. Subjects, thus, filled out a single choice lists such as in Fig. 1, which reflected their joint evaluation. The experimenter remained present in the room during this time to address questions and monitor the experiment. The conversations between subjects were not recorded, we only stored their collective response on the choice lists. After completing the collective task, subjects returned to their individual cubicle and were asked to complete Part 3 without discussing with each other.

### Health state descriptions

Each part consisted of SG and TTO elicitations for the same 3 health states (and 1 practice health state), for which descriptions were obtained from the EQ-5D-5L classification system [46]. The EQ-5D-5L distinguishes between five health domains, i.e., ‘‘mobility”, “self-care”, “usual activities”, “pain/discomfort”, and “anxiety/depression”. Within these domains, this taxonomy uses five health state levels from “no problems” to “extreme problems/unable to”. In EQ-5D nomenclature, health states are represented by 5 digit codes like 22113. This example features as a label for a health state with: slight problems (i.e. level 2) with mobility and self-care, no problems with the usual activities and no pain/discomfort (i.e. level 1), and moderate anxiety/depression (i.e. level 3). To familiarize subjects with the choice list elicitation, they completed a practice elicitation for $Q_{\text{p}}$: 41321 using both SG and TTO choice lists (in Parts 1 and 2). Next, SG and TTO elicitations were completed for three health states, which were relatively mild and ordered monotonically increasing in severity, i.e. each consecutive health state featured more severe problems on at least one domain and was identical otherwise. The following health states were used: 11221 (‘high’), 21222 (‘middle’) and 32322 (‘low’), which we denote $Q_{1} , Q_{2}$ and $Q_{3}$. We selected mild health states to avoid health states that may be considered worse than death, for practical reasons, as such severe health states require a different elicitation procedure [43].

### Data quality

Several checks for data quality were implemented. First, to familiarize subjects with health states $Q_{1} , Q_{2}$ and $Q_{3}$ at the start of this experiment, subjects were required to rate these health states alongside death on a scale between 0 and 100, where 100 represented full health. Second, choice lists did not allow multiple switching points, which traditionally pose a significant problem to this method when it is applied with paper-and-pencil [47]. Third, to test for consistency, SG choice list elicitations were repeated for health state $Q_{1}$ in all Parts (before continuing with TTO). Finally, we were able to determine violations of monotonicity. Given that health states were monotonically increasing in severity, we should obtain $U\left( {Q_{1} } \right) > U\left( {Q_{2} } \right) > U\left( {Q_{3} } \right)$, i.e. monotonically increasing probabilities $p$ accepted in SG and decreasing number of years $T^{\prime}$ in ${\text{FH}}$ for TTO.

### Analyses

We analyzed: (a) decision quality, and (b) decision outcomes (a full transcript of our analyses is available on request). Each of these decision domains was first analyzed by direct comparisons (i.e. *t* tests) at the aggregate level between sessions and conditions. Second, we applied mixed effects regressions in order to (1) determine if collective decisions for SG and TTO influence decision making beyond mere learning, and (2) estimate if collective decision making improves subsequent individual decision making. The former is referred to as a *‘group effect’*, while the latter is referred to as *‘carryover effect’* (see Table 1). For the group effect we compared the group answers in the CDM condition (CDM: G) and the repeated individual answers in the control group (IDM: I2) to their respective baseline. Thus, this comparison consisted of the second time subjects completed SG and TTO weights for both conditions, while individuals in CDM completed this second round in groups. To estimate this group effect, we ran generalized linear mixed effect regressions (LMER) with subject random effects and the following fixed effects included: (1) learning—dummy indicating whether it concerned a first or repeated session, (2) treatment—IDM or CDM, (3) method—SG or TTO and (4) group—interaction term for learning and treatment. The carryover effect was estimated similarly, where we instead compared CDM: I2 and IDM: I2 to their respective baseline. To estimate this carryover effect, we ran a similar LMER, with the same fixed effects included; i.e., (1) learning, (2) treatment, (3) method, and (4) carryover–interaction term for learning and treatment. These analyses were performed with R using the lmerTest package. For the sake of brevity, we will not present full model statistics for the linear mixed-effect analyses, but only report fixed effect estimates (FEE) and standard errors (SE).

## Results

### Decision quality

We analyzed decision quality by determining the effect of collective decision making on our consistency checks and monotonicity of SG and TTO valuations (see Online Appendix C for additional results on precision for SG and TTO, completion times and bargaining weights).

#### Consistency

Consistency on repeated SG choices was adequate for all individual tasks (I1 and I2 for both IDM and CDM), with no significant difference between original and repeated elicitation (*t* tests, *p*’s > 0.07). However, consistency was lower for collective decision making, with significant differences existing between original and repeated decision making (*t* test, *p* < 0.001). Next, we estimated the group effect and carryover effect for consistency (see Table 2). Considering that consistency checks were only applied to SG, we dropped fixed effects for method in both analyses. We found no significant effects in mixed effects regressions.

**Table 2:** Fixed effect estimates (standard errors) for LMER analyses for both group and carryover effects

	Decision quality		Decision outcome	
	Consistency	Monotonicity^a^	QALY weight	Δ(SG-TTO)
Group effect: IDM: I1 vs. I2|CDM: I1 vs G				
Constant	8.87 (2.02)***	1.09 (0.65)^+^	0.50 (0.03)***	0.06 (0.02)***
Learning	− 1.68 (1.25)	0.64 (0.44)	0.04 (0.01)***	0.00 (0.01)
Treatment: CDM	0.15 (2.59)	− 2.75 (1.03)**	− 0.03 (0.03)	0.02 (0.03)
Method: TTO		0.42 (0.28)	− 0.03 (0.01)***	
Group: (learning × treatment)	− 0.74 (1.61)	2.38 (0.86)**	0.01 (0.01)	− 0.01 (0.01)
Health state: middle			0.15 (0.01)***	− 0.04 (0.01)***
Health state: high			0.29 (0.01)***	− 0.08 (0.01)***
Carryover effect: IDM: I1 vs. I2|CDM: I1 vs I2				
Constant	8.87 (1.99)***	1.32 (0.69)^+^	0.51 (0.02)***	0.06 (0.02)*
Learning	− 1.68 (1.22)	0.67 (0.45)	0.04 (0.01)***	0.00 (0.01)
Treatment: CDM	− 1.35 (2.30)	− 0.51 (0.82)	− 0.03 (0.03)	0.01 (0.03)
Method: TTO		0.42 (0.26)	− 0.03 (0.01)***	
Carryover (learning × treatment)	0.76 (1.31)	0.12 (0.55)	0.01 (0.01)	− 0.00 (0.01)
Health state: middle			0.15 (0.01)***	− 0.03 (0.01)***
Health state: high			0.28 (0.01)***	− 0.08 (0.01)***

*,**,***Significance at *p* < 0.05, 0.01 and 0.001, respectively. ^+^Marginal significance at 0.05 < *p* < 0.10

^a^Binomial regression

#### Monotonicity

We determined for each subject to what extent violations of monotonicity occurred per session. A large majority (81–100% depending on session) of our subjects assigned monotonically decreasing QALY weights to all health states. Next, we estimated the group and carryover effect for monotonicity (see Table 2). Subjects were classified as either violators or non-violators; hence, we applied a linear binomial mixed effect model instead of LMER. First, when estimating the group effect, we observe significant effects for: (a) treatment and (b) group. This indicates that: (a) although sampling was random, monotonicity was lower overall for subjects in CDM, and (b) monotonicity increased for collective decisions above and beyond learning. No effects of learning or method were observed. Second, when estimating the carryover effect, we found no significant fixed effects.

### Decision outcome

We analyzed decision outcomes using a similar analytical approach, with a focus on both absolute SG and TTO weights, and the relative differences between these methods.

#### SG and TTO weights

**Fig. 2:**
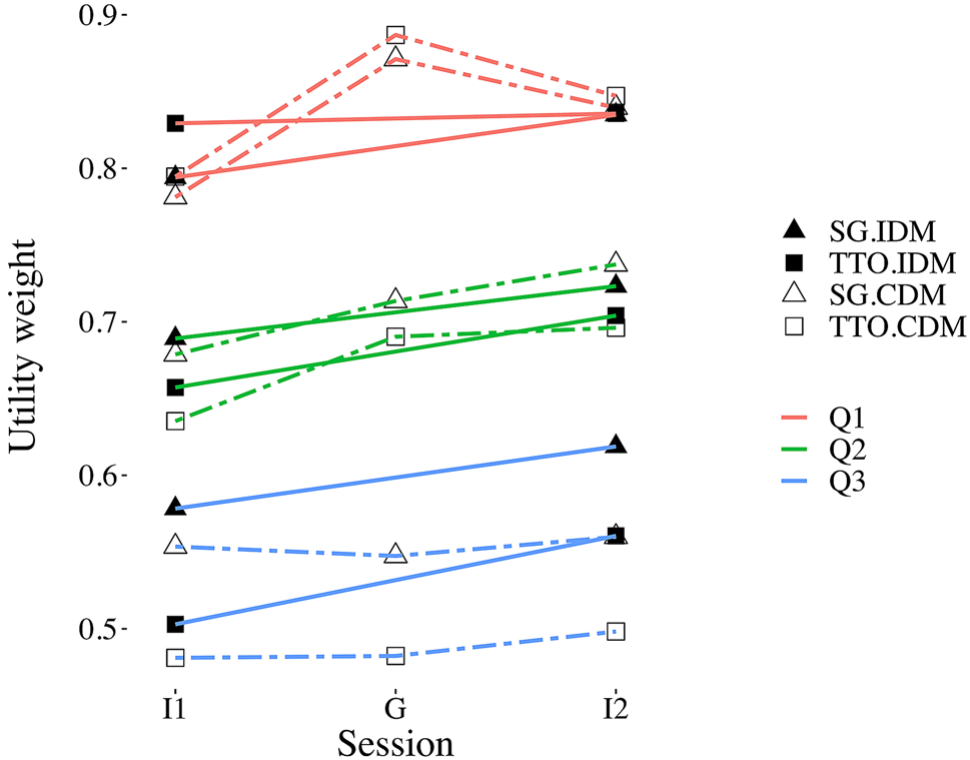
Mean weights split by method (SG vs. TTO), session (I1 vs. G vs. I2), health state ($Q_{1}$ vs. $Q_{2}$ vs. $Q_{3}$) and condition (IDM vs. CDM)

Figure 2 presents the main results on SG and TTO weights. Several trends at the aggregate level can be observed from this figure. First, QALY weights appeared to increase after repetition, with significant within-subjects increases for 9 out of 18 subsequent measurements (all *p*’s < 0.049). For example, for subjects in the IDM condition mean TTO and SG weights for $Q_{3}$ increased from 0.50 and 0.58 in I1 to 0.56 and 0.62 in I2, i.e. 0.06 and 0.04, respectively (see Fig. 2 for the differences for all other measurements). Pooled across all measurements and subjects, each repeated measurement increased QALY weights by 0.03. Next, we estimated the carryover and group effect on the QALY weights, where we ran models with health state included as fixed effect. For both these approaches, we found a significant effect for (a) learning, (b) method and (c) health state dummies. These effects indicate that (a) repetition increases QALY weights, (b) TTO weights were lower and (c) the more severe health states received lower QALY weights. No effect of treatment, group or carryover was observed, indicating that the increase in QALY weights observed on aggregate appears not to be related to collective decisions.

#### Difference between SG and TTO

Next, to test if the difference between SG and TTO reduced as a result of collective decision making we compared the SG-TTO gap per session and health state (denoted ΔSG-TTO). We found consistent evidence of higher weights for SG than TTO in health state $Q_{3}$ (paired *t* tests, all *p*’s < 0.011). For example, for subjects in the IDM condition the mean SG-TTO gap was 0.08 for I1 and 0.06 for I2. However, we found no strong evidence for health state $Q_{2}$ (only significant for CDM-I2, paired *t* test, *p* < 0.01) and $Q_{1}$ (paired *t* tests, all *p*’s > 0.11). We observed a positive SG-TTO gap for baseline measurements (CDM/IDM-I1) pooled across health states with a size of 0.03 (significantly larger than 0, *t* test, *p* < 0.001), suggesting that on average a difference existed between SG and TTO at baseline. Next, we applied our analytical approach to estimate group or carryover effects on this difference between SG and TTO (see Table 2). Only fixed effects for health states were significant, indicating that the difference between SG and TTO increased for more severe health states, and was unaffected by learning or collective decisions.

## Discussion

In this study, we report the first experimental test of the effects of collective decision making on QALY weights. Collective decision making did not appear to have a systematic effect on quality of decisions for SG and TTO; no effects were found for consistency and the initially low monotonicity became up to par with individual decisions. Furthermore, we did find an effect of collective decision making with regard to outcomes for SG and TTO: we found that QALY weights increased, both for collective decisions and for individual decisions. More sophisticated analyses indicated that this increase was only related to learning (and not to facets of collective decisions such as deliberation or bargaining), i.e. repetition increased SG and TTO weights (both in groups and individually). This trend of increased QALY weights for repetition is in accordance with earlier work by Augestad et al. [33]. Given that student samples in some cases have been found to yield low SG and TTO weights (e.g. [10]), this learning effect could be seen as beneficial as it realized a movement towards QALY weights representative of the general population, obtained by a more comprehensive elicitation procedure, shorter SG and TTO durations, and a general public sample [48]. As expected, we replicated the typical SG-TTO gap at baseline [4–6], although this gap was less apparent for the least severe health state. Perhaps this lack of SG-TTO gap for the mildest health state results from a ceiling effect (as QALY weights were close to 1.00). Importantly, we find that the SG-TTO gap was unaffected by collective decision making or learning, and no carryover effects were observed.

This study adds to the evidence base on collective decision making (mostly studies using monetary outcomes). In agreement with the mixed findings of those studies, we do not find a substantial beneficial effect of collective decisions for SG and TTO. However, earlier work on collective decisions for monetary choice suggested that groups discount the future less [11]. Because discounting has a negative effect on TTO values [22], less discounting in the group treatment would cause higher TTO values. Hence, our results could suggest that discounting of health outcomes is not affected by collective decision making; an alternative explanation would be that both discounting and loss aversion decrease in group tasks, which would neutralize each other [22]. Our results also indicate that collective decision making does not alleviate the typical gap between SG and TTO, which is also partially explained as a result of discounting [22, 49]. Future research could therefore obtain separate measurements of discounting and loss aversion (and possibly also other traits such as scale compatibility and probability weighting) for health outcomes to test these possibilities.

Our results confirm findings by Krabbe et al. [27], who find only small differences between collective and individual valuation for SG and TTO using an anonymous voting procedure, and that SG–TTO gaps are unaffected by collective decision making [27]. Furthermore, our findings are in accordance with earlier non-experimental studies on deliberation in TTO or SG, which generally finds that deliberation has little to no effect on QALY weights [25, 26, 50]. Hence, it appears that deciding collectively has no added benefit beyond providing respondents with opportunities for learning. Our findings, thus, suggest that this procedure could be relevant for obtaining nationally representative value sets in settings where providing ample opportunity for learning is too costly or otherwise infeasible.

As in most experiments on the effects of collective decision making in the economic literature, the use of a student sample can be considered a drawback of this study. Obviously, students differ from the general population in a number of ways (so does any particular sub-sample used in empirical work). This is a common criticism of laboratory experiments¸ as any experiment using a non-representative sample will generate questions of external validity. To this end, in experimental economics, usually a distinction is made between experiments aimed at *measurement* and experiments aimed at documenting *treatment effects* [51]. One can question the representativeness of our measurements, as individual QALY weights are typically lower compared to those in the general population [48]. However, we believe it is not as straightforward to question external validity of the treatment effects (i.e. within-subject learning effects and between-subjects group effects), unless one explicitly assumes that these causal processes occur differently for our subject pool than for the general population. For one thing, students are likely to be younger, healthier and higher educated than the general population, and hence, the finding of a substantial learning effect in our student sample may suggest that the inclusion of a sufficient number of practice rounds in health state valuation will be necessary for a sample representative of the general public. We have no reason to expect that deliberation and bargaining are likely to occur differently for students as opposed to the general population. Nonetheless, a potential problem is that our sample consisted of students exclusively, of whom it is likely that they had similar views on length and quality of life (and thus relatively few opportunities to influence each other). Hence, we believe future work should study if in less homogenous dyads (e.g. doctor/patient or student/retiree) the effects of collective decision-making on QALY weights are more prominent.

To conclude, our work suggests that collective decision making does not appear to yield an effect for health state valuation. As in earlier work [27], the difference between SG and TTO does not disappear when moving from an individual to a collective task, which suggests that collective decision making does not help to reduce bias in SG and TTO [22]. Therefore, other solutions for alleviating these confounding effects, such as more elaborate instructions, practice rounds and correction mechanisms [10] should be considered if one aims to correct for these biases.

## Electronic supplementary material

Below is the link to the electronic supplementary material.

Supplementary material 1 (DOCX 144 kb)

## Footnotes

1 We will use the term ‘biases’ to refer to phenomena that yield violations of the linear QALY model and EU, the models that are used to calculate SG and TTO weights in practice.

2 SG and TTO weights can be derived in more general models, which can account for some of the biases driving the differences between SG and TTO (see [10]). However, such derivations are beyond the scope of this paper.

3 Sample size was informed by earlier studies on collective decision making in the economic literature. For example, the average sample size for all empirical studies on collective decision making cited in our Introduction is *n* = 103, with an average of *n =* 40 observations for groups (with sizes ranging from 2 to 5). Ethical approval was received from Erasmus Research Institute of Management’s Internal Review Board, Section Experiments.

4 Often, health state valuation studies use a 10-year duration [43]. For this student sample a 10-year duration followed by death would obviously entail a large decrease in life expectancy. It has been found that such a mismatch between durations in health state valuation and expectations about length of life may lead to biases in health state valuation [44, 45]. Hence, we chose a much longer duration, more closely matched to our respondents’ actual life expectancy.
