# Supplementary material for: A comparison of individual and collective decision making for standard gamble and time trade-off
Source: Eur J Health Econ. 2020 Jan 4;21(3):465–73. doi: 10.1007/s10198-019-01155-x (PMC7188732; doi:10.1007/s10198-019-01155-x)
Supplement: Supplementary file 1 — Supplementary material 1 (DOCX 144 kb) [file 10198_2019_1155_MOESM1_ESM.docx]

**Appendix A: Example instruction for Part 1 (Practice health state)**

In part 1, you have to perform 2 tasks.

Task 1

Suppose you have to choose between 2 possible life scenarios, which are referred to as Alternative A and Alternative B. In Alternative A, you will be certain to live 50 more years in the indicated health state, after which you will die. For example, suppose the health state is as given below:

Your health state (P):

-You have severe problems in walking about

-You have no problems in washing or dressing yourself

-You have moderate problems doing your usual activities (e.g. work, study, housework, family or leisure activities)

-You have slight pain or discomfort

-You are not anxious or depressed

 If you choose Alternative B, you are taking a gamble. On the one hand, you have the chance (X%) of living 50 more years in full health (i.e. no problems on any dimension), after which you will die, but on the other hand, you have a chance (100-X %) of dying within a week.

 The task consists of a number of lists of choices between the two alternatives. In every list, Alternative A remains the same, but Alternative B varies.

As you move down the list, Alternative B becomes more attractive, and in some row, you will probably switch from Alternative A to Alternative B. If so, you will also choose Alternative B in all rows below that one, because in these Alternative B is more attractive. Similarly, if you choose Alternative A in a given row, you will also choose Alternative A in all rows above that one, because in these Alternative B is less attractive. The computer takes this into account and automatically selects Alternative B for all rows below the one where you choose Alternative B and Alternative A for all rows above the one where you choose Alternative A.

There are no right or wrong answers, we are only interested in your choices.

You can change your choices as often as you like. Once you are satisfied with your choices, click the “OK” button. Then you can no longer change your choices and you receive the next choice list.

Please now choose the alternative you prefer in each row. If you are ready, you get a prompt on your screen. At that moment, please read the instruction of Task 2 on the next page.

Instructions Task 2

Again, suppose you have to choose between 2 possible life scenarios, which are referred to as Alternative A and Alternative B.

In Alternative A, you will live 50 more years in the indicated health state, after which you will die. For example, suppose the health state is as given below:

 Your health state (P):

-You have severe problems in walking about

-You have no problems in washing or dressing yourself

-You have moderate problems doing your usual activities (e.g. work, study, housework, family or leisure activities)

-You have slight pain or discomfort

-You are not anxious or depressed

If you choose Alternative B, you will live X more years in full health (i.e. no problems on any dimension), after which you will die.

 Please choose the alternative you prefer in each row. This procedure is similar as in Task 1.

**Appendix B: Screenshots of the experimental program**

Task 1: Standard Gamble


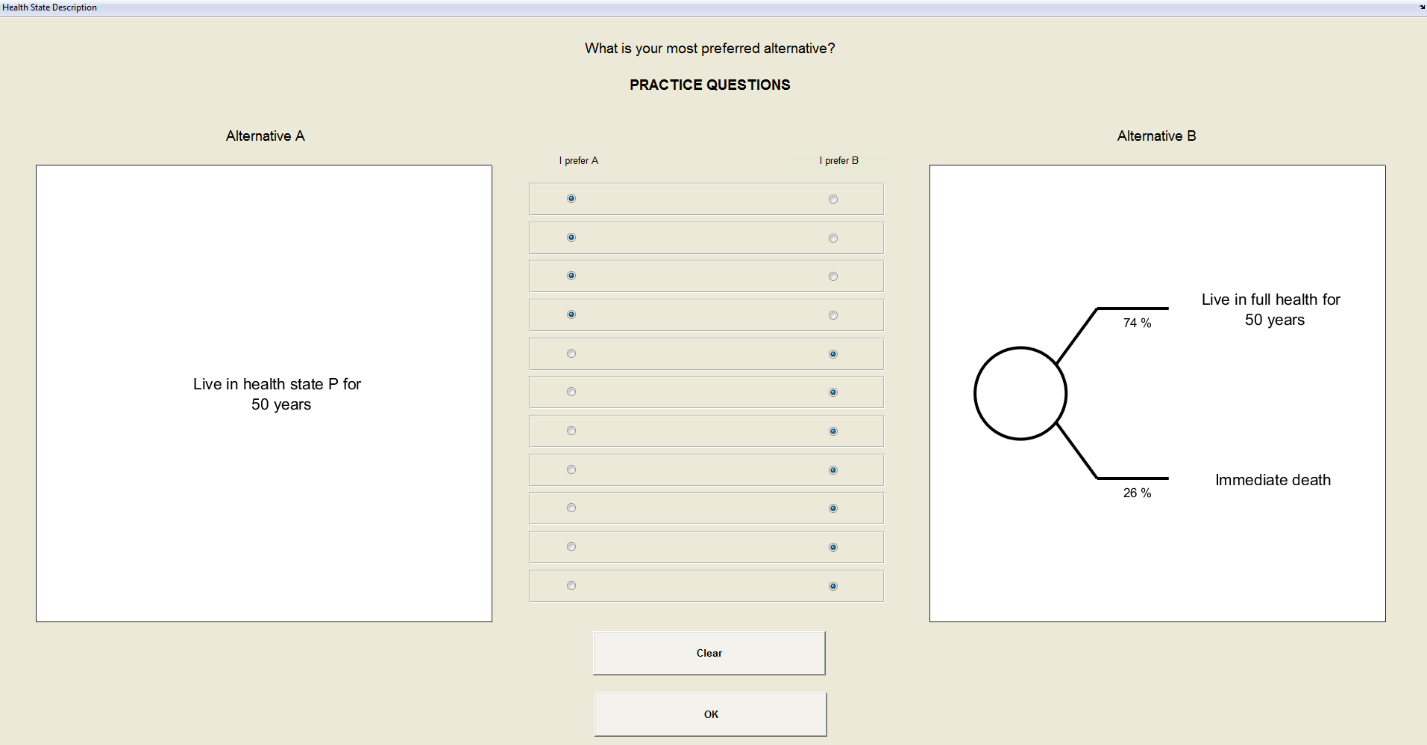


Task 2: Time trade-off


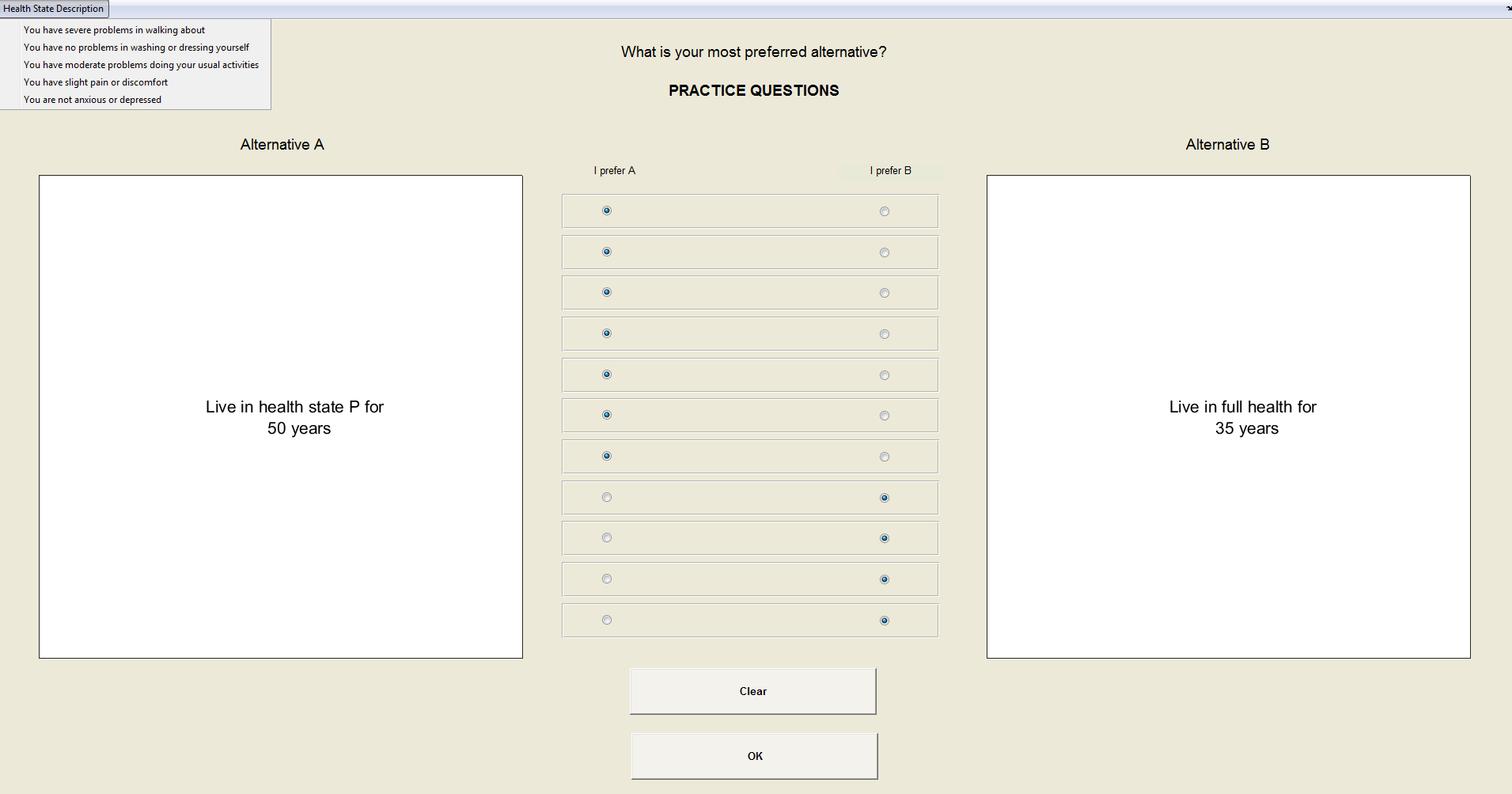


**Appendix C: Additional results on precision, completion time and decision process for SG and TTO**

Two additional elements of quality of decision making were analyzed, the precision of utility weights and the completion time for each elicitation. We also estimated the group and carryover effect for these decision elements, which can be found in Table C1.

**Table C1.** Fixed effect estimates (standard errors) for LMER analyses for both group and carryover effects

|  | **Decision process** | |
| --- | --- | --- |
|  | **Precision** | **Time** |
|  |  |  |
| **Group effect :** IDM: I1 vs. I2 \| CDM: I1 vs G | | |
| Constant | 0.03 (0.01) *** | 72.74 (3.86) *** |
| Learning | -0.004 (0.004) | -15.91 (1.99) *** |
| Treatment | -0.005 (0.009) | -17.37 (4.75) *** |
| Method: TTO | 0.01 (0.002)*** | -12.91 (1.25) *** |
| Group: (Learning*Treatment) | 0.009 (0.005) + | 15.32 (2.55) *** |
| Health state: middle |  | 5.54 (1.53) *** |
| Health state: high |  | 13.55 (1.53) *** |
|  |  |  |
| **Group effect :** IDM: I1 vs. I2 \| CDM: I1 vs G | | |
| Constant | 0.03 (0.01) *** | 76.54 (2.65) *** |
| Learning | -0.004 (0.004) | -19.89 (1.74) *** |
| Treatment | -0.001 (0.009) | 4.49 (4.11) |
| Method: TTO | 0.01 (0.003) *** | -13.65 (1.09) *** |
| Carryover (Learning*Treatment) | 0.006 (0.005) | -6.53 (2.24) ** |
| Health state: middle |  | 6.34 (1.34) *** |
| Health state: high |  | 20.35 (1.34) *** |

**Note:** *,**, and *** represent significance at p < 0.05, 0.01 and 0.001 respectively. + indicates marginal significance at 0.05< p <0.10.

C.1. Precision

Precision was analyzed both between-subjects and within-subjects. For between-subjects comparisons, we apply Morgan-Pittman tests for equality of variances to compare between session variance within-methods. For example, we compare SG weight variance for state $Q_{1}$ between session I1 and session I2. These tests indicated the degree to utility weights were heterogeneous between sessions and health states. For IDM variances were not significantly different between I1 and I2 (Morgan-Pittman tests, all p’s > 0.16). If we repeat these analyses (I1 vs I2) for CDM, we find a significant decrease (Morgan-Pittman tests, all p’s < 0.034) in variance, with the exception of the most severe health state $Q_{3}$ for both SG and TTO (Morgan-Pittman tests, p’s > 0.15). For CDM, we observe significantly smaller variance between the first individual session and group task (Morgan-Pittman tests, all p’s < 0.023). The estimation of fixed group or carryover effects is not possible, as these variance estimates reflect between-subjects heterogeneity. Second, we obtain within-subjects estimates of precision by calculation of variance for utility weights associated with $Q_{1}$, $Q_{2}$ and $Q_{3}$ (see Table C2). These analyses indicate to what extent collective decision making affected dispersion of utility weights for each individual, i.e. if utility weights elicited in each session become more condensed or dispersed. Next, when we applied our analytical approach to estimate for the group effect and carryover on within-subject variance (see Table C1), we observed only a fixed effect of method, implying higher dispersion for TTO compared to SG. We observed no effects of learning, treatment, group or carryover effects of collective decision making.

**Table C2.** Decision quality: Mean within-subjects variance and percentages of subjects satisfying monotonicity for each session

|  | Session 1 | |  | Session 2 | | Session 3 |
| --- | --- | --- | --- | --- | --- | --- |
|  | I1-IDM | I1-CDM |  | I2-IDM | Group | I2-CDM |
| Variance for $Q_{1}$, $Q_{2}$, & $Q_{3}$ |  |  |  |  |  |  |
| SG | 0.024 | 0.030 |  | 0.022 | 0.035 | 0.034 |
| TTO | 0.040 | 0.043 |  | 0.033 | 0.048 | 0.043 |

C.2. Completion time

Completion times were recorded for each session and separately for each health state within each session. Unsurprisingly, for our full sample baseline measurements took longer (5.5 minutes on average) than second individual measurements (little over 3 minutes on average), i.e. repetition decreased time needed for completion (*t*(294) =10.09 , *p* < 0.001). When we focused on subjects in CDM, we observed that group measurements (around 5.5 minutes) took approximately as long as baseline measurement (paired t-test, t(190) = -0.20, p = 0.84). When applying our analytical approach on within-subjects completion times, similar to our analyses on decision outcomes, fixed effects were also obtained for health states separately, to determine if completion times were affected by severity. In turned out that both when estimating the group and carryover effect almost all fixed effects were significant. The only fixed effect that was not significant was that of treatment in the carryover effects model (p=0.28). Collectively, these findings indicated that decision time consistently decreased: from TTO compared to SG, for repeated sessions, for more severe health states. Furthermore, the group and carryover effect indicated that collective decisions took longer, while subsequent individual measurements were completed faster for subjects in CDM.

C.3. Bargaining weights

Finally, we explored the collective decision making process by analyzing decision dynamics within dyads completing the CDM task. We estimated to what extent group QALY weights deviated from QALY weights we observed for the group members at baseline (i.e. I1-CDM). At the aggregate level, a pattern in which the group elicitation falls in-between the two individual estimates is observed most frequently (see Table 3). Such a pattern suggests that a majority of groups reached a consensus somewhere in-between their individual estimates (except for TTO-Q1). Nonetheless, outside consensus group utility weights (lower than min, higher than max) are not uncommon and represent between 28 and 43% of the groups, depending on health state and method. When we investigated within-group consensus (i.e. the proportion of consensus across methods and health states), we observed that groups reach consensus in almost two-thirds of elicitations (64.97%). Only two groups (4%) failed to reach consensus on any elicitation on both SG and TTO. We also found no effect of reaching a consensus or not carrying over into subsequent individual decisions in CDM-I2 (t-tests, all p’s > 0.18).

**Table C3.** Decision process: Location of group utility weight compared to individual weights and median decision weight for high valuators (n = 49).

|  | SG-$Q_{1}$ | SG-$Q_{2}$ | SG-$Q_{3}$ | TTO-$Q_{1}$ | TTO-$Q_{2}$ | TTO-$Q_{3}$ |
| --- | --- | --- | --- | --- | --- | --- |
| Location of utility weight |  |  |  |  |  |  |
| Below the min | 4 | 10 | 11 | 3 | 9 | 8 |
| Above the max | 13 | 10 | 6 | 11 | 12 | 6 |
| At the min | 4 | 2 | 2 | 4 | 2 | 4 |
| At the max | 7 | 3 | 2 | 15 | 3 | 2 |
| In-between | 21 | 24 | 28 | 17 | 23 | 29 |
| Decision weight | 0.89 | 0.64 | 0.43 | 1.00 | 0.72 | 0.44 |

**Note:** Min and max refer to the lowest and highest individual valuation, respectively.

Next, we estimated the decision weight associated with the higher individual QALY weight in a given group, i.e. the high valuator, for a given decision. We obtained this decision weight by assuming that collective decisions were a weighted summation of individual QALY. In other terms, we calculated decision weight $\propto_{H}$ of the high valuator in group QALY weights (GQW), by rearranging the following equation: $GQW= \propto_{H}*{IQW}_{H}+\left( 1-\propto_{H} \right)*{IQW}_{L}$. Here, ${IQW}_{H}$ and ${IQW}_{L}$ reflect baseline QALY weights for the high valuator and their partner who assigned lower utility to that health state, respectively. In this context, if $\propto_{H}$ > 0.5 the high valuator has more weight in decisions, while for $\propto_{H}$ < 0.5 the opposite holds. For the sake of clarity, we removed 6 observations corresponding to the cases where the two individuals’ QALY weights were identical. Table 3 shows that for the best health state ($Q_{1}$), the group tended to follow the individual with the higher utility, whereas the opposite occurred for the worst health state (both for SG and TTO).
